# Exploring the underlying molecular connections: a bioinformatics approach to link melanoma and Parkinson’s disease

**DOI:** 10.3389/fgene.2025.1526018

**Published:** 2025-02-24

**Authors:** Limei Zhang, Dailin Li, Xu Zheng, Moli Wu, Qijun Yao, Haoran Chen, Zhiqiang Ye, Bo Yuan

**Affiliations:** ^1^ Department of Reparative and Reconstructive Surgery, Second Affiliated Hospital of Dalian Medical University, Dalian, Liaoning, China; ^2^ Neurology Department, Central Hospital of Dalian University of Technology, Dalian, Liaoning, China; ^3^ First Affiliated Hospital of Dalian Medical University, Dalian, Liaoning, China; ^4^ College of Basic Medical Science, Dalian Medical University, Dalian, Liaoning, China

**Keywords:** melanoma, Parkinson’s disease, bioinformatics analysis, differentially expressed genes, molecular docking

## Abstract

**Introduction:**

Melanoma, a highly aggressive form of skin cancer, and Parkinson’s disease (PD), a progressive neurodegenerative disorder, have been epidemiologically linked, showing a positive association that suggests a shared etiology. This association implies that individuals with one condition may have an increased risk of developing the other. However, the specific molecular mechanisms underlying this relationship remain unclear. This study aimed to elucidate the molecular mechanisms by conducting a comprehensive comparative analysis of gene expression profiles in both PD and melanoma to identify common differentially expressed genes (DEGs) that may contribute to the pathophysiological overlap between these two conditions.

**Methods:**

We analyzed two independent publicly available genomic datasets to identify overlapping DEGs associated with both PD and melanoma. Regulatory networks, including transcription factors (TFs), DEGs, and microRNAs (miRNAs), were constructed. Protein-protein interaction (PPI) networks were established to identify hub genes. Additionally, we investigated the interplay between PD, melanoma, and immune cell infiltration to uncover potential correlations between the expression levels of hub genes and specific subsets of immune cells. Molecular docking studies were performed to identify potential therapeutic agents targeting the DEGs.

**Results:**

A total of 41 overlapping DEGs were identified, including VSNL1, ATP6V1G2, and DNM1, which were significantly down-regulated in both PD and melanoma patients. These genes play critical roles in biological processes, cellular components, and molecular functions relevant to the pathogenesis of both diseases. VSNL1 is associated with synaptic vesicle fusion and may impact neuronal communication compromised in PD. ATP6V1G2, a subunit of the V-ATPase, is involved in the dysregulated pH homeostasis observed in melanoma. DNM1, a key player in vesicle trafficking, may influence aberrant cellular transport processes in both diseases. Regulatory and PPI networks revealed potential hub genes and their interactions. Molecular docking studies identified retinoic acid as a potential therapeutic agent targeting VSNL1, ATP6V1G2, and DNM1.

**Discussion:**

Our study provides insights into the shared molecular characteristics of PD and melanoma, identifying potential biomarkers for early diagnosis and prognosis and revealing new therapeutic targets. The discovery of retinoic acid as a promising therapeutic agent represents a significant step forward in drug development and treatment strategies for these diseases. This comprehensive analysis enhances our understanding of the intricate molecular mechanisms underlying the association between PD and melanoma, paving the way for further research and therapeutic advancements. The findings hold the promise of improved diagnosis, prognosis, and personalized treatment strategies for individuals affected by these debilitating diseases.

## 1 Introduction

Melanoma, a highly aggressive form of skin cancer, and Parkinson’s disease (PD), a progressive neurodegenerative disorder, have been reported to exhibit a positive association. However, the underlying molecular mechanisms remain unclear. Recent scientific evidence has highlighted an increased risk of occurrences for one disease in patients suffering from the other, sparking significant interest among researchers (Bose et al., 2018). Melanoma is one of the most immunogenic tumors, and its progression is significantly influenced by the host’s immune system. The interplay between melanoma cells and immune cells within the tumor microenvironment plays a crucial role in tumor biology, including proliferation, differentiation, and progression (Snyman et al., 2024). It accounts for the majority of skin cancer-related deaths globally and exhibits high metastatic potential (Stătescu et al., 2023). On the other hand, PD primarily affects the substantia nigra pars compacta region of the brain, leading to the degeneration of dopaminergic neurons. This loss of neurons results in motor impairments, including tremors, bradykinesia, and postural instability. The immune system also plays a role in PD, where neuroinflammation contributes to the degeneration of dopaminergic neurons. This inflammation involves the activation of microglia and astrocytes, which release cytokines and other inflammatory mediators that can exacerbate the disease (Morris et al., 2024; Camargo et al., 2024). Several recent studies have already shed light on potential connections between melanoma and PD through different avenues. For instance, both diseases exhibit dysregulation of various cellular processes, including protein degradation pathways (Sun et al., 2023; K et al., 2022), oxidative stress response (Subramaniam and Chesselet, 2013; Tsoi et al., 2018), and immune modulation (Huang and Zappasodi, 2022; Tansey et al., 2022). Additionally, common genetic variants have been identified in genes associated with both melanoma and PD, suggesting shared susceptibility loci (Lubbe et al., 2016). Additionally, common genetic variants have been identified in genes associated with both melanoma and PD. Some examples include variants in the PDCD1 gene, such as the PD1.5 and PD1.7 SNVs, which have been linked to both conditions (Boutros et al., 2024). These variants are thought to influence immune responses and may contribute to the shared susceptibility observed in melanoma and PD. Furthermore, the SLC45A2 gene, which is associated with melanoma, has also been implicated in PD through its role in melanin production and oxidative stress management. This body of work provides a foundation for our hypothesis that there may be common molecular pathways and mechanisms underlying both conditions.

The field of biomedical research has witnessed a remarkable transformation in recent years due to the emergence of high-throughput sequencing technologies and the vast repositories of genomic data. The application of bioinformatics, which combines biology, computer science, and statistics, has provided researchers with powerful tools to analyze large-scale biological datasets and uncover hidden patterns, associations, and potential mechanisms. This integrative approach allows us to explore the complex interactions between genes, proteins, and other biomolecules, thereby unraveling the molecular underpinnings of various diseases.

Understanding the shared mechanisms underlying melanoma and PD holds immense value from both scientific and clinical perspectives. The findings of this study have the potential to drive future research and clinical applications. Firstly, it provides an opportunity to identify common molecular signatures that can serve as potential biomarkers for early detection and diagnosis. Early identification of these diseases is critical, as timely intervention significantly improves patient outcomes. Secondly, elucidating shared pathways could lead to the discovery of new therapeutic targets for both melanoma and PD. Many existing drugs target specific molecules or pathways, and the identification of shared targets may allow for repurposing of approved drugs or the development of novel targeted therapies. Moreover, a deeper understanding of the overlapping mechanisms may aid in the development of preventive strategies, potentially reducing the incidence and burden of both diseases.

It is important to note that while previous studies have suggested an increased risk for one disease in patients with the other, there is no clear evidence of comorbidity between melanoma and PD. This study aims to explore the potential shared molecular mechanisms between these two conditions using bioinformatics analysis of publicly available genomic datasets. By integrating data from diverse sources, such as gene expression profiles, protein-protein interaction networks, and pathway enrichment analyses, we seek to identify key genes, pathways, and regulatory mechanisms that are commonly dysregulated in both diseases. Our analysis will involve mining large datasets to extract meaningful biological insights, ultimately contributing to a deeper understanding of the pathogenesis of melanoma and PD.

## 2 Methods

### 2.1 Data source and identification of differentially expressed genes (DEGs)

The dataset GSE8397, sourced from the Gene Expression Omnibus (GEO) database (https://www.ncbi.nlm.nih.gov/geo/), focuses on PD and leverages microarray technology to explore gene expression variances in the substantia nigra (SN). Specifically, the dataset includes *Postmortem* brain tissue samples from PD and control cases (substantia nigra, split into medial and lateral portions, and frontal cortex). 15 samples of medial parkinsonian SN, 9 samples of lateral parkinsonian SN, 8 medial nigra control samples and 7 lateral nigra control samples. Furthermore, data from the GSE46517 dataset, which investigates gene expression distinctions between melanoma tumor tissues (n = 104) and normal skin samples (n = 7), were also obtained from the GEO database. This dataset encompasses both male and female patients. The analysis of these datasets involved utilizing the R Project, with LIMMA and DESeq2 serving as tools for standardizing the data and performing the analysis of differential gene expression. Subsequent visualization of the DEGs in the datasets was accomplished through the creation of volcano plots and heatmaps in R. The statistical significance threshold was established at |log2(fold change)| > 1 and a FDR-adjusted p-value < 0.05.

### 2.2 KEGG and GO enrichment analysis

We have chosen to filter out genes with TPM values below a threshold of 1 in at least 75% of the samples. The 41 identified DEGs were subjected to KEGG (Kanehisa et al., 2021) pathway enrichment analysis and Gene Ontology (GO) enrichment analysis using the DAVID database (https://davidbioinformatics.nih.gov/) (Dennis et al., 2003). The top 10 most relevant pathways were selected for KEGG analysis, as well as the top 10 for GO analysis. Enriched pathways were identified based on a false discovery rate (FDR) threshold of less than 0.05, serving as the cut-off criterion for significance.

### 2.3 Construction of TFs-DEGs-miRNAs regulation network

To investigate the connections between DEGs, Transcription Factors (TFs), and microRNAs (miRNAs), we utilized the JASPAR website set a threshold of a minimum matrix similarity score of 0.85 (https://jaspar.elixir.no) to predict 60 TFs that interact with each gene and the miRTarBase website (https://mirtarbase.cuhk.edu.cn) to predict 763 miRNAs associated with the DEGs. Subsequently, the regulatory network encompassing TFs-DEGs-miRNAs was visualized using Cytoscape. Furthermore, to enhance the analysis of our findings, we integrated biomolecular interaction networks with the high-throughput expression data and other molecular states. For this purpose, we accessed protein-protein and functional interaction networks from the STRING database (https://string-db.org/) for the 41 hub genes identified in our study. To visualize these networks and better understand their relationships, we utilized Cytoscape software (Shannon et al., 2003).

### 2.4 Hub genes identification

To explore the network of hub genes, we utilized the CytoHubb Plugin within Cytoscape, which provides several methodologies for identifying key nodes in biological networks and understanding their connections with other genes. Our study aimed to identify the top 10 hub genes using various algorithms such as betweenness, closeness, degree, EPC, and MNC ranking methods. These algorithms helped us highlight critical genes within the network. In the resulting network, we visually represented the hub nodes by color-coding them based on their significance. The highest score was denoted by red, while the lowest was indicated by yellow. To further analyze the identified hub genes, we applied Venn diagram analysis to determine the common interacting hub genes among the top 10 genes generated by each ranking method. Through this analysis, we identified a set of common interacting hub genes, including NFKBIA, STXBP1, VSNL1, SNCA, MYO5A, TAGLN3, ATP6V1G2, and DNM1.

### 2.5 Immune cell infiltration and correlation analysis

The immunological infiltrations of immune cells were assessed using the CIBERSORT algorithm (Newman et al., 2015) to examine the immune microenvironment in patients with PD and melanoma. The expression profiles of 22 immune infiltrating cell types were illustrated through box plots. Furthermore, the ggplot2 package was utilized to visualize the relationship between different immune cell types and VSNL1, ATP6V1G2, and DNM1 through Spearman correlation analysis.

### 2.6 Identification of diagnostic genes

Receiver Operating Characteristic (ROC) curve analysis was conducted, and the Area Under the Curve (AUC) values were computed using the pROC package in R software to assess the predictive capabilities of VSNL1, ATP6V1G2, and DNM1 in datasets GSE8397 and GSE46517. To investigate the dynamic variations in diagnostic genes associated with PD and melanoma, ROC curves of these diagnostic genes in PD and control groups, as well as in melanoma and normal patients, were analyzed. AUC > 0.8 indicates good discrimination. The biomarker or test is more effective at separating the disease group from the control group. It suggests that the biomarker has a reasonably high accuracy in predicting the presence of the disease, although some false positives and false negatives may still occur.

### 2.7 Prediction of potential therapeutic drugs and molecular docking

The DSigDB database (Yoo et al., 2015) was utilized to predict potential therapeutic drugs targeting hub genes. The ligand structures were imported into the AutoDock Vina (Eberhardt et al., 2021) environment. AutoDockTools was used to charge and hydrogenate the ligand small molecule, and then rotate the small molecule around any Angle to remove the binding memory. The initial protein structures (VSNL1, ATP6V1G2, and DNM1) were obtained from the Protein Data Bank (PDB) and processed using PyMOL. Water molecules were removed from the structures to facilitate subsequent processing. Hydrogen atoms were added to the protein to account for missing hydrogens, and the protonation states of ionizable residues were adjusted to reflect physiological conditions. The protein structures were validated using PyMOL to assess their quality and reliability. The grid box is centered to cover the domains of each protein and accommodate free molecular motion, and the docking pocket is set as a 30 Å × 30 Å × 30 Å square pocket with a lattice distance of 0.05 nm. Results of docking were then visualized using molecular graphics software PyMOL. The ranking of the docking poses was performed using the scoring function provided by AutoDock Vina, with lower values indicating stronger binding.

### 2.8 Statistical analysis

Data analysis was performed using R version 4.2.0 software and GraphPad Prism version 8.0.1. The data are presented as normalized expression values using the limma package’s normalizeBetweenArrays function. Group comparisons were conducted using unpaired Student’s t-test, comparing PD patients to controls and melanoma samples to normal skin samples. Receiver operating characteristic (ROC) curves were generated to assess the area under the curve (AUC) values and evaluate the predictive performance of the analyzed data. A statistical significance threshold of p < 0.05 was used to determine the significance of the results.

## 3 Results

### 3.1 Common DEGs identified in patients with PD and melanoma

**FIGURE 1:**
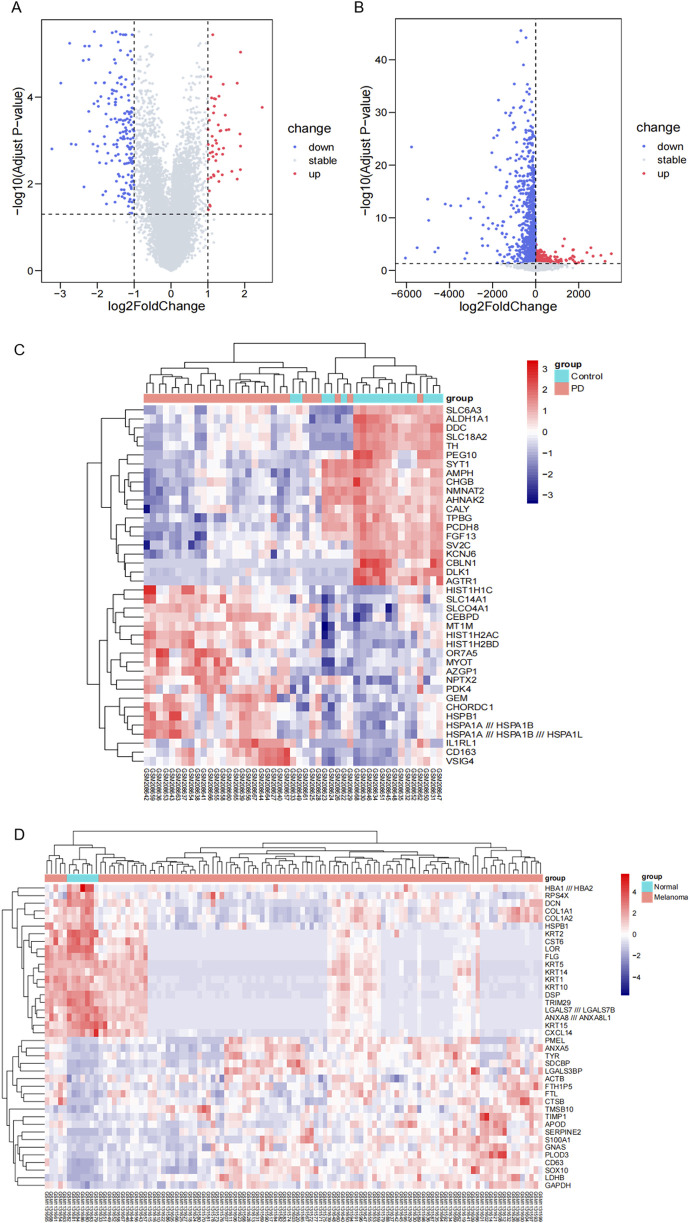
The identification of DEGs. **(A)** Volcano plot displaying DEGs in the GSE8397 dataset. Upregulated genes are highlighted in red, while downregulated genes are shown in blue. **(B)** Volcano plot presenting DEGs in the GSE46517 dataset. Upregulated genes are denoted in red, and downregulated genes in blue. **(C)** Heat maps illustrating the top 40 DEGs with the most prominent changes in the GSE8397 dataset. **(D)** Heat maps depicting the top 20 DEGs with the most significant alterations in the GSE46517 dataset.

We conducted an analysis of gene expression in two datasets, GSE8397 and GSE46517, to investigate common DEGs associated with Parkinson’s disease and melanoma. In GSE8397, we observed a downregulation of 174 genes, while 13,291 genes remained stable, and 51 genes were upregulated (Figure 1A). To provide a visual representation of the most significant changes, we present heat maps displaying the expression patterns of the top 40 DEGs in Figure 1C. Similarly, in the GSE46517 dataset, we identified 1,458 downregulated genes, 11,367 stable genes, and 691 upregulated genes (Figure 1B). The heat maps in Figure 1D illustrate the expression patterns of the top 40 DEGs with the most significant changes.

### 3.2 Functional enrichment analysis of DEGs

**FIGURE 2:**
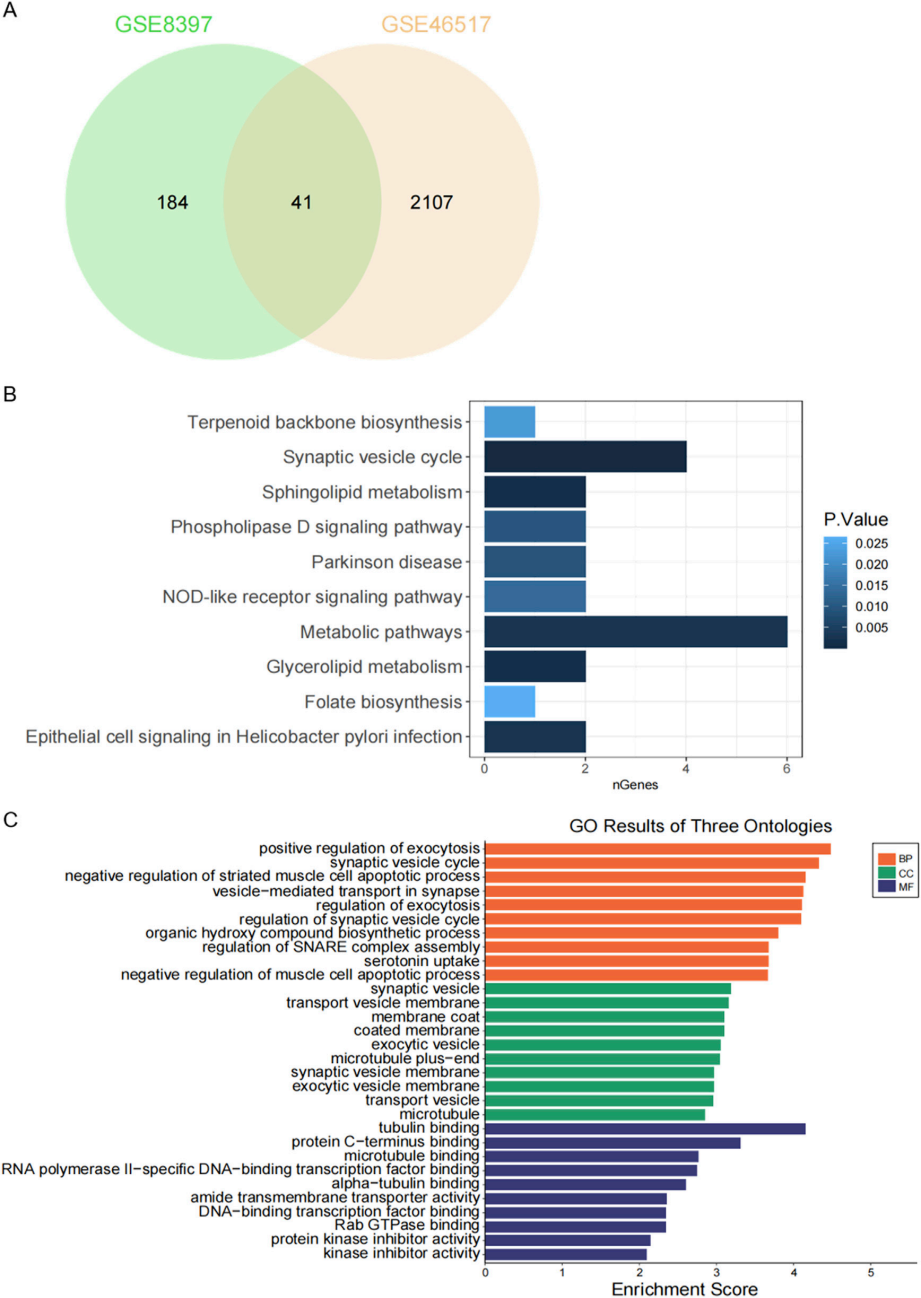
Functional enrichment analysis of DEGs. **(A)** Venn diagram showing overlapping DEGs between the two GEO datasets. **(B)** KEGG pathway analysis of 41 shared DEGs. **(C)** Gene Ontology (GO) enrichment analysis of 41 common DEGs.

We identified 41 overlapping genes from the combination of DEGs in GSE8397 and GSE46517 for further investigation (Figure 2A). To gain deeper insights into these DEGs, we conducted functional enrichment analysis. Specifically, we performed KEGG pathway analysis to uncover potential biological pathways associated with the 41 overlapping DEGs. The results revealed a significant enrichment of these genes in the Synaptic Vesicle Cycle and Metabolic Pathways (Figure 2B). Furthermore, we conducted Gene Ontology Biological Process (GO-BP) enrichment analysis, which highlighted the proteins’ involvement in positive regulation of exocytosis, synaptic vesicle cycle, and negative regulation of striated muscle cell apoptotic process. In terms of Gene Ontology Cellular Component (GO-CC), the majority of these proteins were found to be enriched in synaptic vesicle, transport vesicle membrane, and membrane coat. Lastly, Gene Ontology Molecular Function (GO-MF) enrichment analysis indicated that the 41 overlapping DEGs were associated with tubulin binding, protein C-terminus binding, and microtubule binding (Figure 2C).

### 3.3 Analysis of TFs-targets-miRNAs regulating networks

**FIGURE 3:**
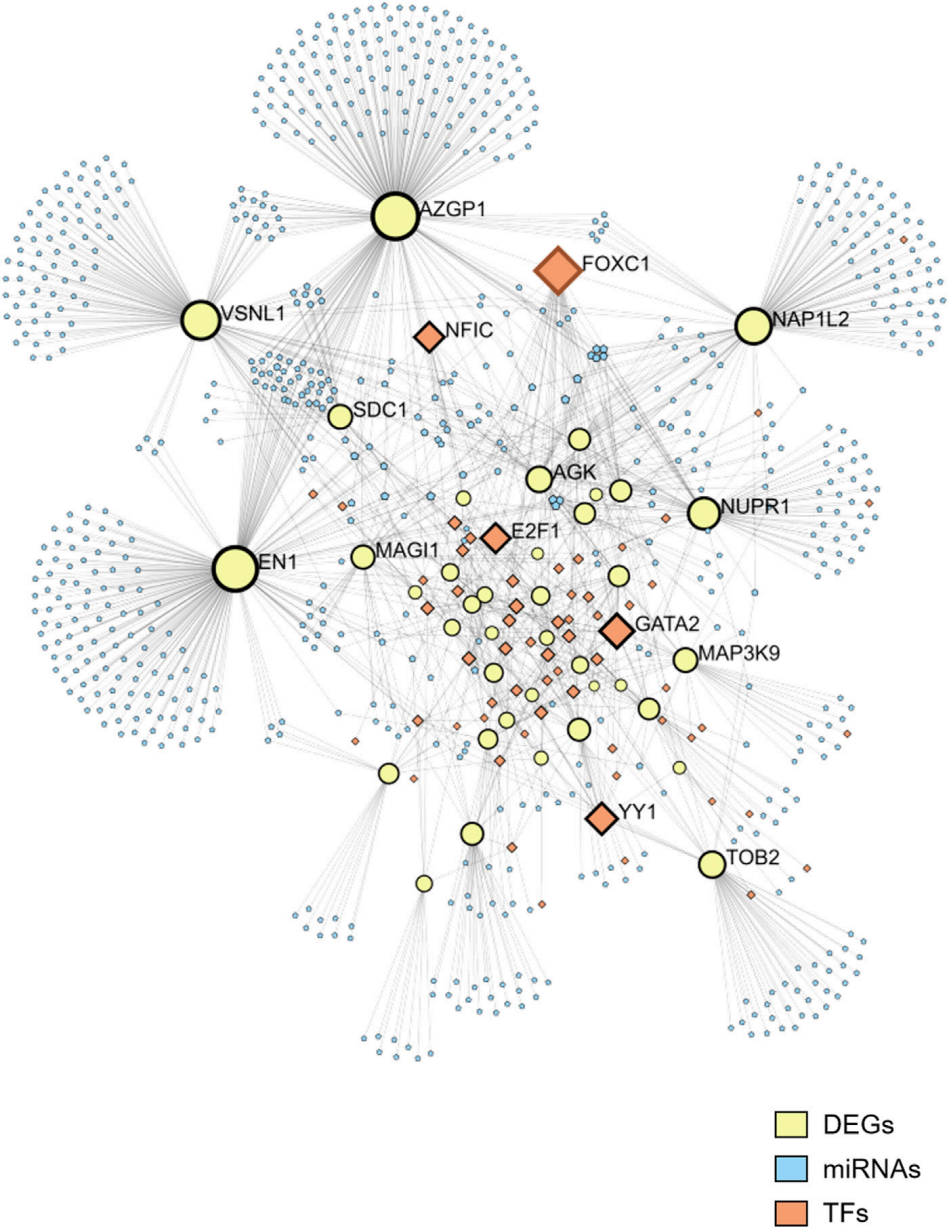
Network of TFs, DEGs, and microRNAs. The network includes 39 DEGs, 60 TFs, and 763 miRNAs. DEGs are represented by yellow nodes, miRNAs by blue nodes, and TFs by orange nodes.

To gain a deeper understanding of the regulatory mechanisms underlying the selected DEGs, as well as the miRNAs targeted by these genes, we utilized JASPAR to predict the TFs of the DEGs and miRTarBase to identify the miRNAs associated with the DEGs. The predictions resulted in a network comprising 39 DEGs, 60 TFs, and 763 miRNAs (Figure 3). Among the top five TFs identified in this network are FOXC1, GATA2, YY1, E2F1, and NFIC. This TF-DEGs-miRNAs network encompasses a total of 862 nodes and 1,370 edges, highlighting the complex interactions between these elements. In the network visualization, DEGs are represented by yellow nodes, miRNAs by blue nodes, and TFs by orange nodes (Figure 3).

### 3.4 Analysis of protein-protein interaction (PPI) networks and identification of hub genes

**FIGURE 4:**
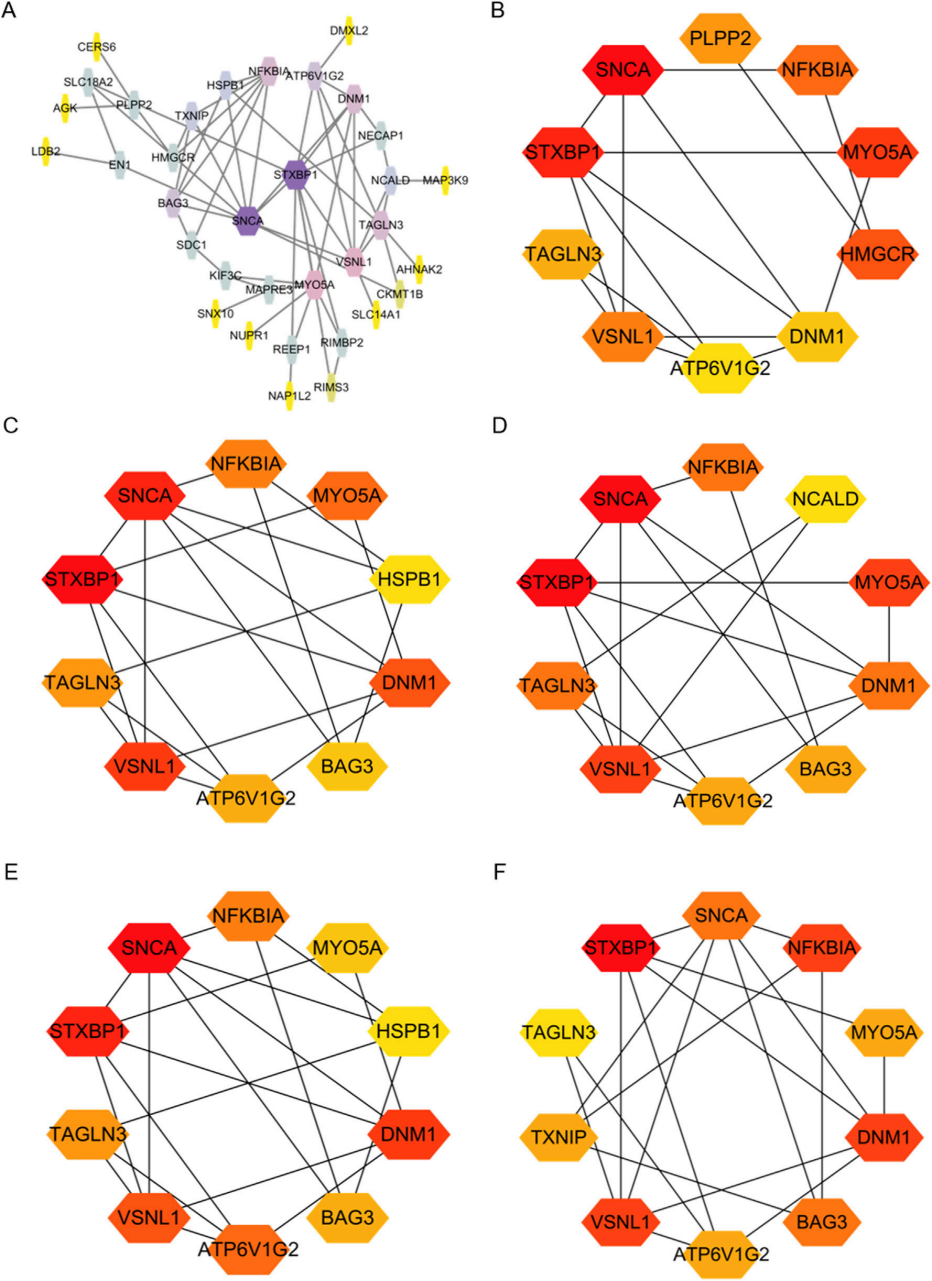
Identification of Hub Genes in overlapping DEGs between the two GEO datasets. **(A)** Protein-Protein Interaction (PPI) network of DEGs constructed using String and Cytoscape. **(B–F)** Hub genes identified using various ranking methods: betweenness, closeness, degree, EPC, and MNC.

**FIGURE 5:**
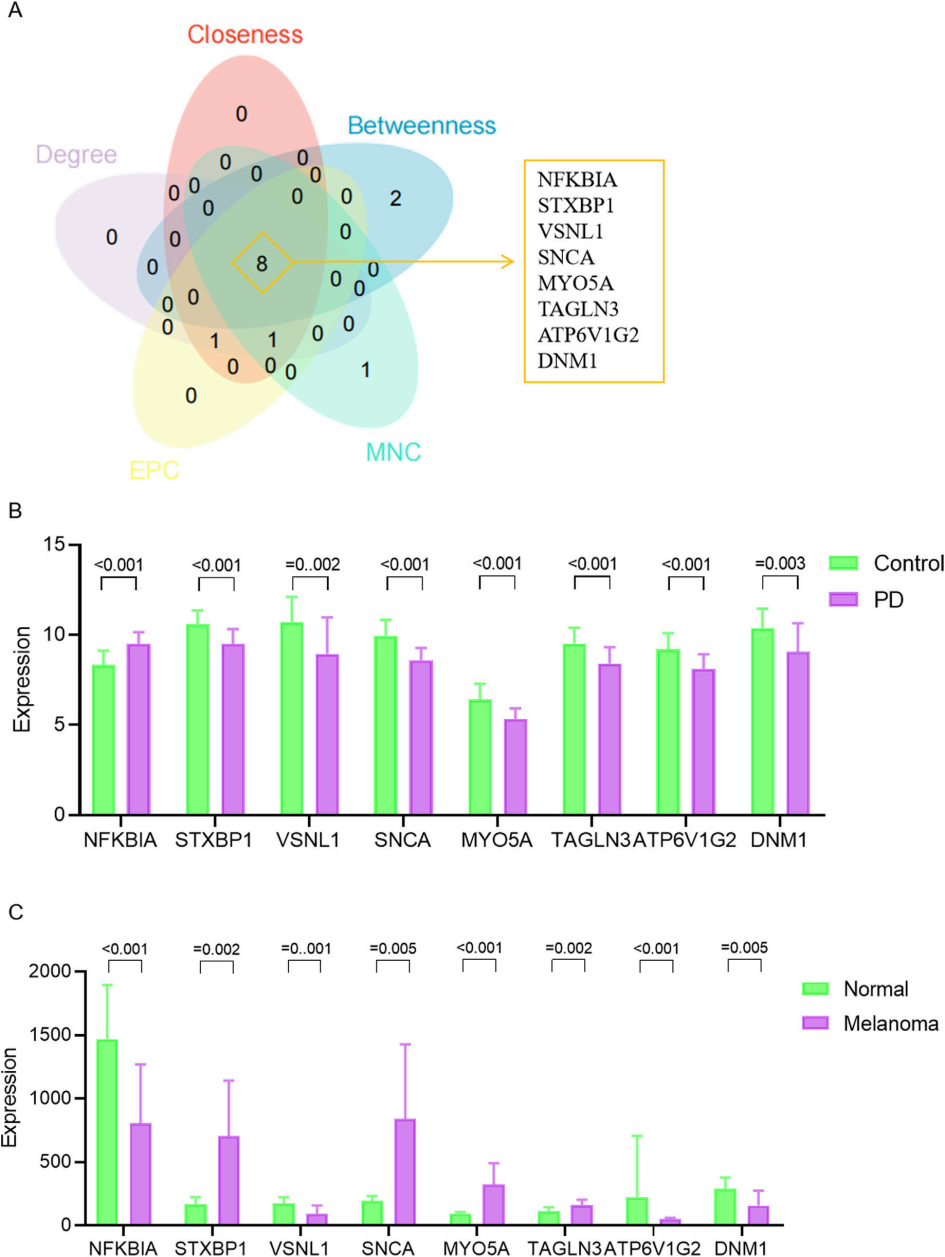
Expression levels of hub genes. **(A)** Venn diagram illustrating hub genes identified across different ranking methods. **(B)** Expression profiles of hub genes in the GSE8397 dataset. **(C)** Expression patterns of hub genes in the GSE46517 dataset.

We constructed a PPI network for the 41 overlapping genes using the STRING database and visualized it using Cytoscape software (Figure 4A). To identify hub genes within this network, we employed the cytoHubba tool in Cytoscape v3.10.0, which employs various ranking methods. Specifically, we employed several ranking methods including betweenness, closeness, degree, edge percolated component (EPC), and maximal neighborhood component (MNC) rankings (Figures 4B–F). Each method provided a list of top-ranked proteins, allowing us to compile a set of potential hub genes. By intersecting the results from these five approaches, we identified NFKBIA, STXBP1, VSNL1, SNCA, MYO5A, TAGLN3, ATP6V1G2, and DNM1 as the common interacting hub genes (Figure 5A). To validate the expression levels of these hub genes in each dataset, we extracted their expression data from the two datasets. Our analysis revealed that among these eight hub genes, only VSNL1, ATP6V1G2, and DNM1 exhibited significant downregulation in both PD patients and melanoma patients (Figures 5B, C).

### 3.5 Association between PD, melanoma, and immune cell infiltration

**FIGURE 6:**
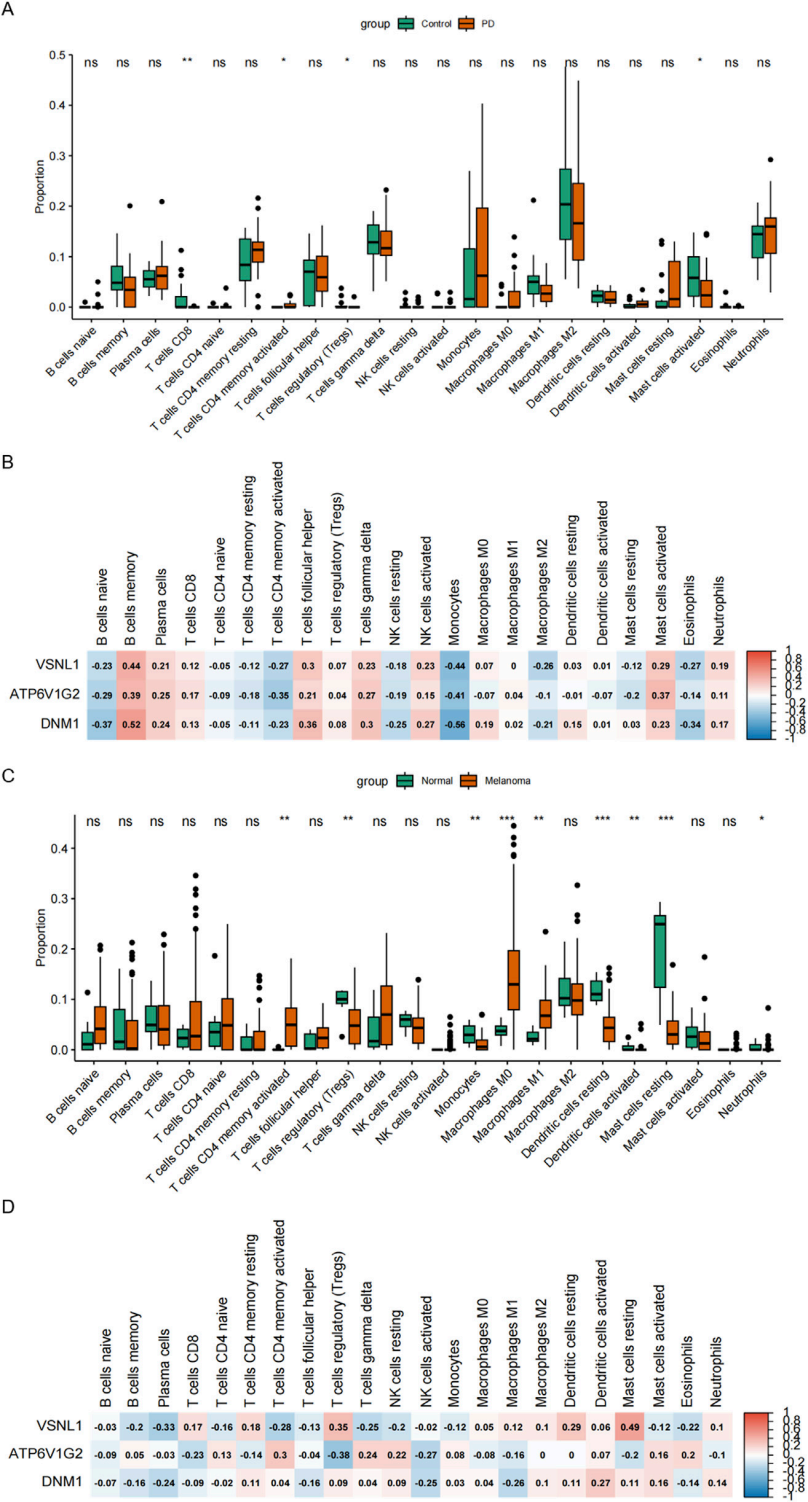
Association of PD and Melanoma with immune infiltration. **(A)** Relative abundance of distinct immune cell subsets in the GSE8397 database. **(B)** Correlation between immune cell infiltration and the expression of VSNL1, ATP6V1G2, and DNM1 in PD. **(C)** Relative abundance of different immune cell subsets in the GSE46517 database. **(D)** Correlation between immune cell infiltration and the expression of VSNL1, ATP6V1G2, and DNM1 in melanoma.

Immune cell infiltration in the substantia nigra of PD patients and *postmortem* brain substantia nigra samples from control cases (including the medial and lateral substantia nigra) was assessed. Monocytes and macrophages were found to constitute a substantial portion of the immune microenvironment in both patient groups. Notably, PD tissues exhibited a higher presence of monocytes compared to controls, although the difference was not statistically significant. Furthermore, the PD group displayed a greater proportion of CD4 memory activated T cells but significantly lower expression levels of activated mast cells compared to the control group (Figure 6A). The roles of VSNL1, ATP6V1G2, and DNM1 genes in the immune microenvironment of PD patients were explored through evaluation of inflammatory cell infiltration. The results indicated a strong negative correlation of these genes with monocytes, a certain negative correlation with CD4-activated T cells, and a certain positive correlation with mast cells, suggesting a close relationship between these proteins and the immune microenvironment in PD patients (Figure 6B). Figure 6C in our analysis using data from the GSE46517 database depicted the relative abundance of different immune cell subsets in the context of melanoma. Notable variations were observed in multiple immune cell populations, including T cells, monocytes, dendritic cells, mast cells, and neutrophils, between the melanoma group and the normal group. Specifically, the melanoma group exhibited significantly lower levels of regulatory T cells, monocytes, resting dendritic cells, and mast cells compared to the control group. Conversely, higher levels of CD4-activated memory T cells and M0 macrophages were observed in the melanoma group. Furthermore, Figure 6D presented the correlation analysis between immune cell infiltration and the expression levels of VSNL1, ATP6V1G2, and DNM1 in melanoma. Among these genes, VSNL1 demonstrated the highest positive correlation with resting mast cells. ATP6V1G2 showed the strongest negative correlation with regulatory T cells. On the other hand, DNM1 exhibited limited correlation with various immune cell types collectively.

### 3.6 Diagnostic values of hub genes

**FIGURE 7:**
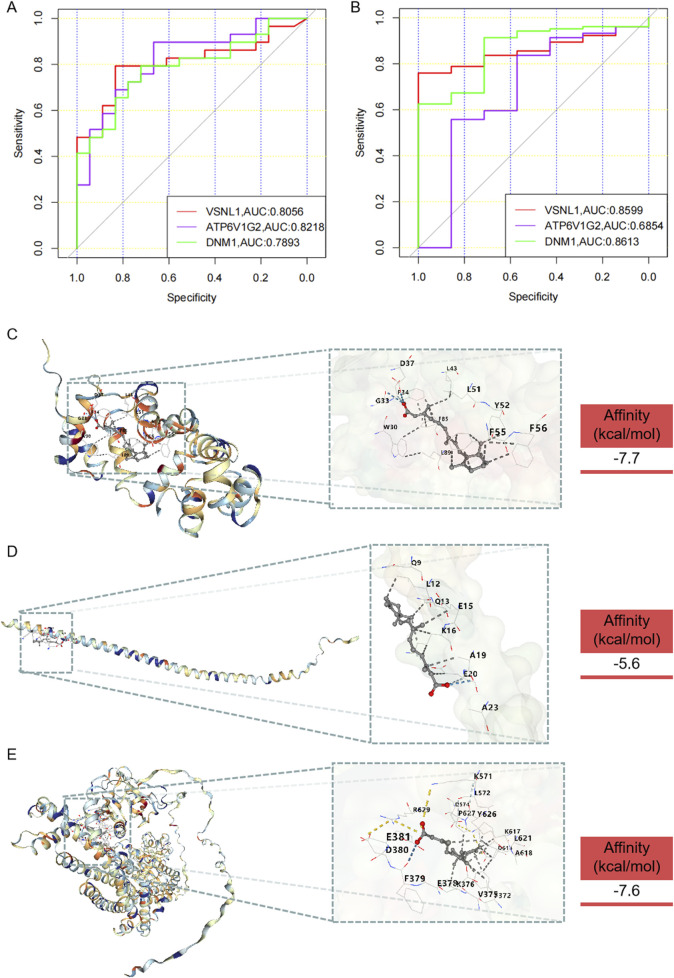
Diagnostic value of hub genes and prediction of potential drugs. **(A)** ROC curve and AUC statistics of the predictive model for PD in the GSE8397 dataset. **(B)** ROC curve and AUC statistics of the predictive model for melanoma in the GSE46517 dataset. Three-dimensional molecular docking diagrams illustrating the binding of retinoic acid with the target proteins: **(C)** VSNL1, **(D)** ATP6V1G2, and **(E)** DNM1.

The diagnostic performance of hub genes was highlighted through the presentation of ROC curves and the corresponding AUC statistics in GSE8397 and GSE46517 datasets (Figures 7A, B). The ROC curve serves as a tool to assess the predictive model’s performance, while the AUC value reflects the overall accuracy of the model in discriminating between PD and Control, as well as between Melanoma and Normal samples. In the GSE8397 dataset, ATP6V1G2 exhibited the highest diagnostic value with an AUC of 0.8218, followed by VSNL1 with an AUC of 0.8056. Furthermore, in GSE8397, both VSNL1 and DNM1 genes displayed AUC values surpassing 0.800, with VSNL1 recording an AUC of 0.8599 and DNM1 with an AUC of 0.8613. These results offer valuable insights into the expression profiles of the hub genes and underscore their potential utility as diagnostic markers for Parkinson’s disease and melanoma.

### 3.7 Molecular docking diagrams of simvastatin with target proteins

In the quest for potential anti-PD and anti-melanoma medications, drugs capable of targeting VSNL1, ATP6V1G2, and DNM1 were identified through screening in the DsigDB database (Supplementary Table S1). The analysis identified retinoic acid and valproic acid as potential therapeutic agents for VSNL1, ATP6V1G2, and DNM1. Although experimentally validated data did not confirm their binding to ATP6V1G2, our results indicated that retinoic acid could successfully bind to VSNL1, ATP6V1G2, and DNM1. However, valproic acid did not exhibit binding affinity towards these proteins (Supplementary Table S2). The molecular docking outcomes are depicted in Figures 7C–E. These findings provide insights into the potential mechanisms of action and therapeutic effects of retinoic acid under the conditions of the study.

## 4 Discussion

Our study holds significant importance in understanding the potential connections between PD and melanoma at the molecular level. The novelty of this study lies in the identification of overlapping DEGs between PD and melanoma, which sheds light on potential shared molecular mechanisms underlying these two conditions. Our hypothesis, grounded in the scientific evidence of increased risk of occurrences, led us to explore the molecular underpinnings that may explain this association.

Our study identified 41 overlapping DEGs in Melanoma and PD, suggesting potential shared molecular mechanisms between these two diseases. Functional enrichment analysis provided deeper insights into the biological processes and pathways associated with these DEGs. These pathways provide valuable insights into the molecular mechanisms that may underlie the shared pathogenesis of these two seemingly disparate diseases. Notably, the Synaptic Vesicle Cycle and Metabolic Pathways were prominently enriched, highlighting the potential convergence of these processes in melanoma and PD (Calafate et al., 2023; Yu et al., 2017). The Synaptic Vesicle Cycle pathway is crucial for neurotransmitter release, a process that is disrupted in PD, leading to motor impairments (Ratnikov et al., 2017; Shao et al., 2021). Our findings suggest that similar dysregulations may be at play in melanoma, potentially affecting neural mechanisms that contribute to tumor behavior. The enrichment of Metabolic Pathways points to the disruption of energy metabolism, a hallmark of PD, and suggests a similar metabolic reprogramming in melanoma that may support its aggressive growth and resistance to therapy. Furthermore, the enrichment analysis highlighted the association of the overlapping DEGs with tubulin binding, protein C-terminus binding, and microtubule binding. Tubulin, a key component of microtubules, plays a critical role in maintaining neuronal structure and function. Altered tubulin dynamics have been observed in both melanoma and PD, suggesting their potential involvement in disease pathology (Kapitein and Hoogenraad, 2015). In PD, disruptions in microtubule dynamics can impair the transport of synaptic vesicles and other organelles, leading to neuronal degeneration. Similarly, in melanoma, altered microtubule dynamics can affect cell division and migration, contributing to tumor progression and metastasis (Higgins et al., 2020; Naren et al., 2023). The identification of these pathways allows us to hypothesize that melanoma and PD may share common molecular vulnerabilities. In melanoma, the dysregulation of pigmentation and immune response pathways is well-documented. Our study extends this by suggesting that these pathways may also be perturbed in PD, potentially contributing to the neuroinflammatory aspects of the disease. Conversely, pathways typically associated with PD, such as those involved in dopamine metabolism and neuronal protection, may play a role in melanoma progression, influencing tumor microenvironment and response to treatment. Additionally, our study identified several transcription factors, such as FOXC1 and GATA2, within the TF-DEGs-miRNAs network. These transcription factors are known to modulate the expression of the hub genes and regulate disease-relevant pathways. For instance, FOXC1 has been implicated in the regulation of cell proliferation and migration in various cancers, including melanoma. GATA2, on the other hand, plays a role in immune cell development and function, which could influence the immune response in both PD and melanoma. By modulating the expression of key hub genes, these transcription factors may contribute to the dysregulation of critical pathways involved in disease pathogenesis (Han et al., 2017; Menendez-Gonzalez et al., 2019; Fang et al., 2019; Høie et al., 1987). Understanding the regulatory interactions between these TFs and the identified DEGs may shed light on the underlying mechanisms contributing to PD and melanoma.

Furthermore, the construction of a PPI network enabled the identification of hub genes within the overlapping DEGs. NFKBIA, STXBP1, VSNL1, SNCA, MYO5A, TAGLN3, ATP6V1G2, and DNM1 emerged as common interacting hub genes. Among these, VSNL1, ATP6V1G2, and DNM1 exhibited significant downregulation in both PD patients and melanoma patients, suggests a common molecular mechanism underlying these two conditions. VSNL1 proteins have been implicated in various cellular processes, including calcium signaling, apoptosis, and neuroprotection (Tage et al., 2023; Spilker et al., 2002). Several studies have reported altered VSNL1 expression in the brain tissues of PD patients, suggesting its involvement in PD pathogenesis (Lin et al., 2015; Groblewska et al., 2015). ATP6V1G2 is a component of the vacuolar ATPase (Qi et al., 2022). Dysregulation of ATP6V1G2 has been associated with neurodegenerative diseases (Li et al., 2020). DNM1 is involved in the process of endocytosis and vesicle formation (Xie et al., 2020; von Spiczak et al., 2017). The diagnostic values of these genes were evaluated through ROC curve analysis, and the results demonstrated their potential utility as diagnostic markers for PD and melanoma. The high AUC values obtained for ATP6V1G2, VSNL1, and DNM1 indicate their potential as reliable biomarkers for the early detection and diagnosis of these diseases. Our analysis of immune cell infiltration in Parkinson’s disease (PD) and melanoma has shed light on the immune microenvironment specific to these conditions. In the context of PD, we observed that monocytes and macrophages were notably prevalent within the immune microenvironment, and there was a significant increase in the frequency of activated CD4 memory T cells when compared to non-diseased states. This observation is consistent with existing literature that emphasizes the role of both innate and adaptive immune components in the pathogenesis of PD (Weiss et al., 2022; Dhanwani et al., 2022). Moreover, our research has identified a role for the genes VSNL1, ATP6V1G2, and DNM1 within the immune microenvironment of PD patients. The inverse relationship between these genes and the levels of monocytes and CD4-activated T cells, coupled with a positive association with the density of mast cells, suggests that these genes may be instrumental in regulating the presence and activity of certain immune cell populations in PD. In the context of melanoma, our findings reveal a unique pattern of association between these genes and immune cell infiltration, underscoring their potential role in the immunological response to tumors.

Lastly, molecular docking analysis provided insights into potential therapeutic agents targeting the hub genes. Retinoic acid was found to have binding affinity for VSNL1, ATP6V1G2, and DNM1, indicating its potential as a therapeutic agent for PD and melanoma. Retinoic acid, a derivative of vitamin A, has been extensively studied for its therapeutic potential in various diseases, including cancer and neurodegenerative disorders (Brown, 2023; Szutowicz et al., 2015). Previous studies have shown the involvement of retinoic acid in regulating gene expression, cell differentiation, and apoptosis pathways (Mezquita and Mezquita, 2019; Zhou et al., 2016). The docking results provide valuable insights into the potential mechanisms of action of retinoic acid on VSNL1, ATP6V1G2, and DNM1. By interacting with these proteins, retinoic acid may regulate their activities and downstream signalling pathways, thereby influencing the pathogenesis of PD and melanoma. Further studies are warranted to investigate the specific molecular interactions between retinoic acid and these target proteins and to elucidate the downstream effects of such interactions.

However, it is crucial to acknowledge the limitations of this study. Firstly, the scope of our analysis was constrained by the selection of a limited dataset from the Gene Expression Omnibus (GEO) database. Although the chosen datasets, GSE8397 and GSE46517, are well-characterized and provide valuable gene expression data, the generalizability of our findings may be limited due to the restricted sample size and demographic representation. This constraint affects the breadth of our conclusions and suggests that our results should be interpreted with caution until further validated with larger and more diverse datasets. The analysis was based on publicly available gene expression datasets, which may introduce potential biases and confounding factors. Furthermore, while our study suggests an increased risk for one disease in patients with the other, it does not provide direct evidence of comorbidity between melanoma and PD. The functional enrichment analysis and network analysis relied on computational algorithms and databases, which are subject to inherent limitations and potential errors. Experimental validation of the identified pathways and interactions is necessary to confirm their biological relevance. Lastly, the exploration of environmental factors, lifestyle choices, and genetic predispositions in the context of melanoma and PD comorbidity is an area ripe for investigation. Understanding how these factors interact with the molecular mechanisms we have identified could offer new avenues for prevention and personalized treatment strategies.

In conclusion, our analysis identified common DEGs associated with PD and melanoma, shedding light on potential shared molecular pathways and mechanisms underlying these diseases. The functional enrichment analysis highlighted the involvement of synaptic processes, vesicular trafficking, and microtubule dynamics in PD and melanoma. The identification of hub genes and their correlations with immune cell infiltration emphasized the potential interplay between these genes and the immune system. Lastly, the diagnostic values of the hub genes and the potential therapeutic targets revealed through molecular docking analysis provide a foundation for future research and clinical applications in the field of PD and melanoma.

## Data Availability

The original contributions presented in the study are included in the article/Supplementary Material, further inquiries can be directed to the corresponding author.
